# Terpenes and Essential Oils in Pharmaceutics: Applications as Therapeutic Agents and Penetration Enhancers with Advanced Delivery Systems for Improved Stability and Bioavailability

**DOI:** 10.3390/pharmaceutics17060793

**Published:** 2025-06-18

**Authors:** Greta Kaspute, Tatjana Ivaskiene, Arunas Ramanavicius, Simonas Ramanavicius, Urte Prentice

**Affiliations:** 1 State Research Institute Center for Physical Sciences and Technology, Sauletekio Av. 3, LT-10257 Vilnius, Lithuania; greta.kaspute@ftmc.lt (G.K.); arunas.ramanavicius@chf.vu.lt (A.R.); simonas.ramanavicius@ftmc.lt (S.R.); 2 State Research Institute Centre for Innovative Medicine, Santariskiu St. 5, LT-08410 Vilnius, Lithuania; tatjana.ivaskiene@imcentras.lt; 3 Department of Physical Chemistry, Institute of Chemistry, Faculty of Chemistry and Geosciences, Vilnius University, Naugarduko St. 24, LT-03225 Vilnius, Lithuania; 4 Department of Mechatronics, Robotics and Digital Manufacturing, Faculty of Mechanics, Vilnius Gediminas Technical University, Plytines St. 25, LT-10105 Vilnius, Lithuania

**Keywords:** essential oils, carrier matrix, controlled release, compound delivery, terpenes

## Abstract

This review examines the pharmaceutical applications of essential oils (EOs) and terpenes, highlighting their dual role as therapeutic agents and natural penetration enhancers. These volatile, hydrophobic compounds have well-documented antimicrobial, antioxidant, and anti-inflammatory properties. However, their clinical potential is limited by poor water solubility, high volatility, and sensitivity to environmental factors, including light, heat, and oxygen. To address these challenges, various advanced delivery systems have been developed to enhance stability, bioavailability, and controlled release. These systems not only protect chemical integrity but also exploit these compounds’ abilities to interact with lipid membranes, facilitating the transport of active compounds across biological barriers. Additionally, their inherent antimicrobial properties can contribute to the overall stability of formulations. The review critically examines the incorporation of terpenes and major essential oil (EO) components, such as limonene, linalool, eugenol, α-pinene, and menthol, into delivery systems, assessing their performance in enhancing drug permeability and targeting specific tissues. Current challenges and future directions in terpenes and EO-based delivery strategies are discussed, highlighting their promising role in developing multifunctional and efficient pharmaceutical formulations.

## 1. Introduction

Plant-derived compounds have long been recognized as valuable sources of therapeutic agents, encompassing diverse classes such as terpenoids, glycosides, flavonoids, and polyphenols collectively known as phytoalexins [1]. Among these, essential oils (EOs) represent complex mixtures of volatile aliphatic and cyclic hydrocarbons, predominantly composed of monoterpenoids. These compounds have found wide application in the food, fragrance, and pharmaceutical industries due to their distinctive aromatic profiles and notable antimicrobial activities [2]. Recently, the growing concern over antimicrobial resistance, driven in part by the overuse of conventional antibiotics and antifungal agents, has renewed interest in the antimicrobial potential of terpenes and essential oils (EOs), despite their relatively modest potency compared to synthetic drugs [2]. Beyond their antimicrobial action, EOs also exhibit significant antioxidant properties, effectively scavenging reactive oxygen and nitrogen species (ROS and RNS) that contribute to oxidative stress and the progression of chronic diseases such as cancer, cardiovascular disorders, and arthritis [2]. These broad-spectrum biological activities have positioned essential oils (EOs) as promising candidates for use in both traditional herbal medicine and modern pharmaceutical formulations.

Driven by increasing consumer preference for natural, plant-based therapies over synthetic pharmaceuticals, terpenes, and essential oils (EOs), which are often perceived as safer and more sustainable, are gaining traction as functional and therapeutic ingredients. When used alone or in synergy with other bioactive molecules, terpenes, and essential oils (EOs) demonstrate a range of pharmacological effects, including antimicrobial, anti-inflammatory, and antioxidant actions. Moreover, their ability to modulate the chemical and biological behavior of co-administered compounds underscores the importance of understanding their interactions in complex formulations. However, several physicochemical limitations hinder the clinical translation and broader pharmaceutical application of EOs. These include low water solubility, high volatility, and sensitivity to light, heat, and oxygen [3], as well as poor skin permeation, which is often restricted to superficial layers [4]. To overcome these challenges, recent research has focused on the development of advanced delivery systems (DSs), particularly nano- and lipid-based carriers. Encapsulation of terpenes and essential oils (EOs) in such systems not only enhances their aqueous solubility and chemical stability but also protects them from environmental degradation, reduces toxicity, and improves controlled release and bioavailability [4,5]. Additionally, the use of vegetable-based oils in formulations can further optimize physical properties such as viscosity and heat resistance [4].

The aim of this review is to highlight the potential of essential oils and terpenes in addressing current pharmaceutical challenges by exploring their incorporation into advanced delivery systems. Emphasis is placed on how these systems can enhance the stability, bioavailability, and therapeutic efficacy of essential oils. The review also discusses the emerging role of delivery systems in targeted drug delivery for various disorders, while acknowledging existing barriers to clinical translation, including concerns about toxicity, regulatory limitations, and a lack of comprehensive clinical data.

## 2. Essential Oils as Pharmaceuticals

Classification of EOs is divided into monoterpenes and phenylpropanoids, and each of these two classes includes esters, aldehydes, ketones, alcohols, phenols, and oxides, while hydrocarbon compounds contain terpenes. Due to their diverse and complex compositions, EOs lack a specific single cellular target, with each complex blend exerting multiple cellular effects through their main components [6]. Moreover, EOs are susceptible to degradation when exposed to high temperatures, light, and oxygen [3]. The degradation of EOs depends on their chemical composition, with more hydrogenated compounds like mono- and sesquiterpenes being particularly susceptible to oxidation.

**Figure 1:**
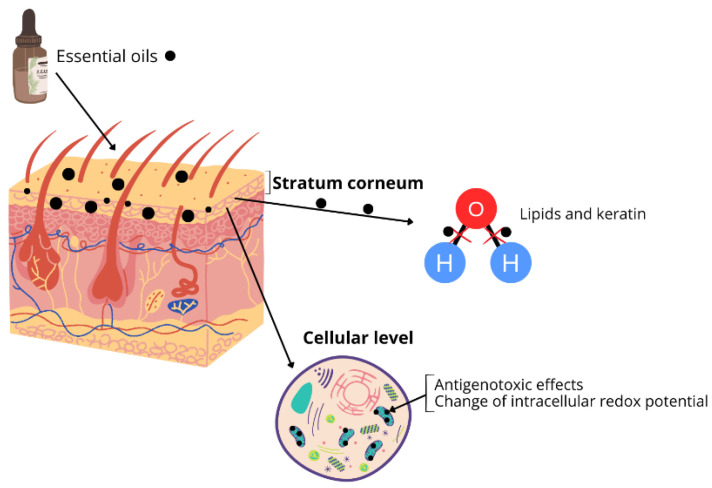
Essential oils penetration through the skin.

Another challenge related to EO application is limited penetration due to their physicochemical properties. The chemical structure of the constituents in EOs plays a significant role in the process of compound penetration through the skin. EOs primarily penetrate the outermost layers of the skin, enhancing epidermal water balance through a “filmogenic” process [4]. Mechanisms involve altering the fluidity of the stratum corneum (SC) membrane through the actions of EOs, which break hydrogen bonds and induce modifications in the state, conformation, and structure of both SC lipids and keratin [4]. Recent studies indicate that EOs can act as prooxidants within eukaryotic cells, affecting organelles such as mitochondria and inner cell membranes. These oils induce antigenotoxic effects and change intracellular redox potential and mitochondrial dysfunction [7] (Figure 1). Understanding these biological and physicochemical properties is essential, as they directly influence the design, efficacy, and safety of essential oils when incorporated into drug delivery systems.

Hydrophilic compounds tend to show improved transdermal absorption with terpenes containing polar functional groups, while hydrocarbon terpenes are more effective in enhancing the absorption of lipophilic compounds, potentially due to differences in thermodynamic activity within gels [8,9]. In subsequent sections, the most common terpenes and their usage cases will be discussed in detail.

### 2.1. Linalool Usage as a Pharmaceutical

Linalool, an acyclic monoterpene tertiary alcohol, is widely distributed across various plant species, including the *Lamiaceae*, *Lauraceae*, and *Apiaceae* families [10]. Linalool has a small molecular weight and a hydroxyl group, which affect polarity. However, the hydrocarbon-based apolar structure of linalool leads to low solubility in water and high solubility in various organic solvents such as alcohol, chloroform, ether, and propylene glycol [10].

The encapsulation of linalool enhances its stability and augments its antioxidant activity, which is crucial for more effective treatment of dermatological conditions [11]. Additionally, recent studies suggest enhancing linalool’s bioavailability and addressing its volatility and poor water solubility while complexing with β-cyclodextrins. The supramolecular structure of β-cyclodextrins features a hydrophilic outer surface and a hydrophobic inner cavity. This configuration allows cyclodextrins to encapsulate lipophilic compounds like linalool, improving water solubility [10]. Incorporating linalool within β-cyclodextrin enhances its bioavailability and antihypertensive efficacy, suggesting a potential novel pharmaceutical formulation for hypertension treatment in upcoming applications [12].

For example, incorporating linalool into DSs for cancer therapy could elevate its anticancer effects while allowing for further modification to target cancer cells and selectively minimize toxicity to normal cells. Nanotechnology-based approaches in drug delivery research offer innovative design features that can enhance the in vivo efficacy of linalool, presenting promising opportunities for its utilization not only in pharmaceuticals as active pharmaceutical ingredients or adjuvants but also in cosmetics [10].

### 2.2. α-Pinene Usage as a Pharmaceutical

α-Pinene, a bicyclic monoterpene, is commonly found in coniferous trees and is a major turpentine component. Due to its minty odor and pine scent, it has been extensively used as a fragrance and flavor ingredient in various essential oils, fruits, vegetables, and herbs [13]. α-Pinene use is limited due to its volatility, low aqueous solubility, and chemical instability. To address these challenges, α-pinene has been encapsulated in conventional liposomes and drug-in-cyclodextrin-in-liposomes systems. The complex of α-pinene combined with hydroxypropyl-β-cyclodextrin improves its solubilization, with optimal conditions achieved at a specific molar ratio [14].

Another study aimed to develop and optimize a self-nano-emulsifying DS of α-pinene and evaluate its in vivo anti-Parkinson’s activity. Various excipient components were screened for miscibility with α-pinene to determine the optimized combination, followed by assessments of self-nano emulsification, thermodynamic stability, dilution robustness, optical clarity, viscosity, and conductivity. Two optimized formulations, composed of Anise oil, Tween 80, and Transcutol-HP oil, were selected, showing improved in vitro drug release profiles compared to free α-pinene suspension. Additionally, the system demonstrated enhanced behavioral activities in rodent models of Parkinson’s disease in vivo [15].

### 2.3. Cineole Usage as Pharmaceutical

1,8-cineole, a natural monoterpene, can serve as a chemical penetrant for transdermal drug delivery, transporting both lipophilic and hydrophilic drugs. For less lipophilic drugs, 1,8-cineole demonstrates notable osmotic-enhancing properties, suggesting its potential to enhance drug permeation. Due to its permeability and low toxicity, 1,8-cineole holds promise for further development in transdermal DSs and could potentially serve as a candidate for brain transport applications [16].

**Figure 2:**
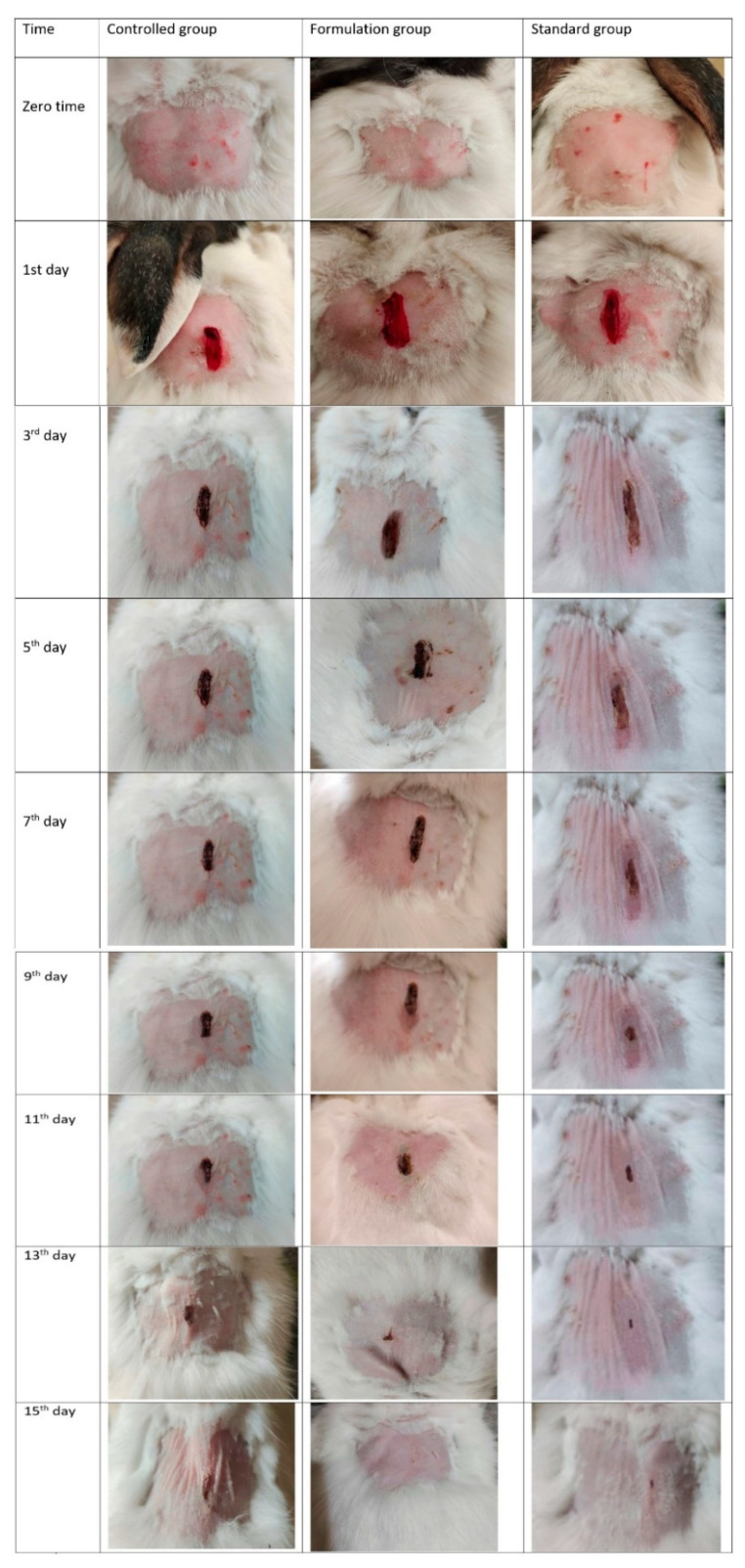
Contraction of wounds in control group, group treated with F5, and group treated with commercial product. F5 is the nanoemulsion containing eucalyptol:tween80:span-60:propulene glycol:black seed oil mixed as 8:30:7.5:13:15 [18].

An optimized self-microemulsifying DS containing 1,8-cineole effectively alleviated lipopolysaccharide (LPS)-induced endothelial injury in Kunming mice. The novel 1,8-cineole delivery system demonstrated significant inhibition of inflammatory cytokines and the attenuation of LPS-induced vascular endothelial injury, potentially mediated through modulation of the NF-κB and peroxisome proliferator-activated receptor γ signaling pathways, suggesting its potential as a therapeutic agent for inflammatory cardiovascular diseases [17]. Nanoemulgels loaded with 1,8-cineole showed favorable characteristics, including pH stability, homogeneity, and acceptable droplet size. In both in vitro (a Franz diffusion cell) and in vivo (rabbit) evaluations, nano emulgels demonstrated significant wound contraction compared to standard and control groups, suggesting their potential as an effective wound-healing alternative [18] (Figure 2).

### 2.4. General Trends in Essential Oil Usage as Pharmaceuticals

The low solubility in water [4] and the exhibition of high oil/water partition coefficients leads to limited production of EOs, restricting their incorporation into hydrophilic products. Their lipid-soluble components enable them to traverse the blood–brain barrier and interact with fluids surrounding the brain [4]. The intense odor impacts sensory properties and might diminish acceptance of the final product [3]. To address stability and stemming issues, the incorporation of EOs into nano-formulations such as nano-emulsions or nano-capsules presents a promising approach, enhancing the physicochemical stability, prolonging their persistence, optimizing the release of active components at the target site, and potentially augmenting their in situ bioactivity [19]. These challenges can be solved by integrating EOs into nanometer-scale surfactant structures, offering a potential solution to enhance their stability and applicability in various formulations [3].

## 3. Design of Novel Essential Oil Delivery Systems

The stability issues of EOs are associated with volatility, oxidation, photosensitivity, heat, humidity, pH, and ion sensitivity. Well-developed DSs should effectively stabilize EOs. Polysaccharides are often chosen as carrier materials due to their safety, versatility, and abundant sources, aligning with the biomimetic concept by mimicking the structure of plant tissue [20]. Nanocarriers can target specific organs and disorders; however, commercialization of them has not been finalized, i.e., toxicity safety has not been granted in clinical studies [21].

Thus, chemical permeation enhancers (CPEs) hold considerable promise for transdermal DSs [16]. The enhanced activity of CPEs is associated with side effects, limiting their progress in transdermal compound delivery. However, advancements in analytical techniques, synthetic chemistry, and combinatorial studies have led to potentially effective CPEs being used in transdermal compound delivery [22]. Biomimetic carrier technology creates emulsions by combining oil, water, and amphiphilic molecules, mimicking natural secretion tissues’ protective mechanisms. This process forms emulsion droplets dispersed within the medium, with EOs encapsulated in the droplets and shielded by an interfacial film, offering protection against volatility and environmental factors [20].

### Main Systems for Essential Oil Delivery

Natural compounds often have a low absorption capacity due to their inability to cross lipid membranes, primarily because of their large molecular size, which leads to reduced bioavailability and efficacy. Additionally, their high systemic clearance demands frequent applications or higher doses, diminishing their therapeutic effectiveness [23]. Encapsulation plays a vital role in protecting and delivering unstable or sensitive compounds, with extensive applications in the pharmaceutical industry. With growing interest, innovative encapsulation strategies continue to advance these applications, enriching both functionality and palatability [24]. Various delivery systems, including lipid-based nanoparticles (NPs), polymeric NPs, dendrimers, inorganic NPs, capsules, hydrogels, oil in water emulsions, and liposomes, can be used to deliver EOs (Table 1) [25,26]. Among these, polymeric NPs and liposomes have been most widely tested due to their biocompatibility, biodegradability, and ease of functionalization [25]. Release systems enhance compound solubility, stability, targeted delivery, bioavailability, and action duration, while potentially delaying compound resistance when combined with natural compounds, offering promising avenues for treating diseases with low response to conventional therapies [23].

Despite the abovementioned delivery systems’ advantages, challenges such as rapid elimination by the reticuloendothelial system, potential toxicity, inflammation, and tissue damage persist. Furthermore, these systems must meet stringent regulatory requirements, including characterization, compound release control, stability, scalability, and cost-effectiveness [25]. For example, liposomal DSs have low compound loading, poor stability, high production costs, and potential toxic side effects; hydrogel DSs are highly dependent on the internal microenvironment; and polymer micelle has long-term safety concerns and limitations in clinical application, which pose significant challenges [27].

**Table 1:** Types of EO delivery systems: advantages and limitations.

Type of DS	Advantages	Limitations	Examples
Emulsions	Emulsions can be water-in-oil (W/O) or oil-in-water (O/W), based on phase location. Micro and nanoemulsions of EOs show improved stability, controlled release, and enhanced antioxidant and antimicrobial properties [26,28]. The absence of surfactants in these systems minimizes environmental impact, making them ideal for utilizing the bioactivity of EOs [29].	The main challenge lies in the poor biopolymer properties and potential flavor impacts from high concentrations of EO [30]. Nanoemulsions with low oil content face challenges in preparation and require fillers, binders, or emulsifiers to enhance viscosity [31].	O/W cinnamon and black pepper emulsions [32], nanoemulsion of eucalyptus oil, Tween 80, and water [33], nanoemulsions of *Cymbopogon flexuosus* [34].
Capsules	Capsules exhibit improved antioxidant and antimicrobial activities, enhanced stability, and controlled release of entrapped EOs [26]. This DS increases bioavailability by adhering to mucous membranes, protects EOs from hydrolysis and oxidation, reduces toxicity and volatility, and enables the targeted delivery of therapeutic doses, improving patient compliance [5].	The main limitations of capsules are volatility and degradation, low encapsulation efficiency, poor release control, and environmental sensitivity.	EO delivery systems based on capsules consisting of zeolites to improve chemical and physical EOs properties [35].
Liposomes	Liposomes offer low toxicity, biocompatibility, non-immunogenicity, enhanced antimicrobial and antioxidant activities, and targeted delivery [26,27].	The use of liposomes is limited by high costs, low compound loading, and poor stability [26,27].	Liposomes loaded with Barije (*Ferula gummosa*) EO were evaluated for their physical properties and antibacterial effects against *Escherichia coli* O157:H7 [36].
Hydrogels	Encapsulating EOs in hydrogels enhances their stability and biological activity [37].	It heavily depends on the internal microenvironment [27]. In addition, there is limited data on stability, safety, long-term bioactivity, and in vivo studies [37].	Semi-solid poly(vinyl alcohol) hydrogels with ginger EO encapsulated in chitosan NPs show promise for wound management applications [38]. Alginate–soy protein isolate complex beads encapsulating thyme EO are designed for targeted intestinal delivery [39].
Solid lipid NPs (SNLs)	SNLs offer better protection, controlled release, and cost-effective formation compared to liposomes and emulsions [26,40].	SNLs exhibit poor stability, aggregation, low encapsulation load, and potential instability under acidic conditions and during storage [26,40,41].	*Zataria multiflora* EO-loaded solid lipid NPs demonstrated antifungal activity under in vitro conditions [42]. Chitosan/polyvinyl alcohol hydrogels loaded with EOs of *Origanum vulgare* and *Thymus vulgaris* in solid lipid NPs effectively controlled the growth of *Botrytis cinerea* and *Penicillium expansum* [43].
Inorganic NPs	EOs and NPs affect gut processes like inflammation, oxidative stress, metabolite synthesis, and microbiota balance [44]. Inorganic NPs have good bioavailability and low toxic side effects tolerance [27]	Potential toxicity depending on dose and exposure, requiring consideration of synergistic and antagonistic interactions [44].	Silver NPs and lavender oil for a synergistic antibacterial effect [45].
Nanostructured lipid carriers (NLCs)	NLCs were developed to overcome SNLs’ limitations, offering smaller size, higher loading capacity, and preventing crystal formation and expulsion [26,41].	SLNs and NLCs require high temperatures for lipid melting, risking degradation of heat-sensitive EO components, and have low stability under acidic conditions and high production costs at an industrial scale [26,46].	NLCs containing 10% *w*/*v* lipid and 10% *w*/*v* EOs (lavender, rosemary, peppermint) show potential for sustainable insect pest control [47]. Red sacaca EO-loaded nanostructured lipid carriers, optimized by factorial design, were evaluated for cytotoxicity and cellular reactive oxygen species levels [48].

## 4. Essential Oils as Penetration Enhancers

To investigate the penetration-enhancing effect of EOs, the efficacy of the whole oil with individual compounds isolated from it should be compared. In vitro study of permeation facilitation of donepezil hydrochloride using oil extracted from *Ledum palustre* L. var. *angustum* N. Busch identified cuminaldehyde (5.72% of the oil composition), which enhances donepezil hydrochloride penetration, demonstrating twice the efficacy of the oil alone. However, other terpenes present in the oil, such as sabinene (33.40%), 4-terpineol (20.33%), and p-cymene (18.31%), exhibited minimal to no efficacy when tested individually [49].

**Figure 3:**
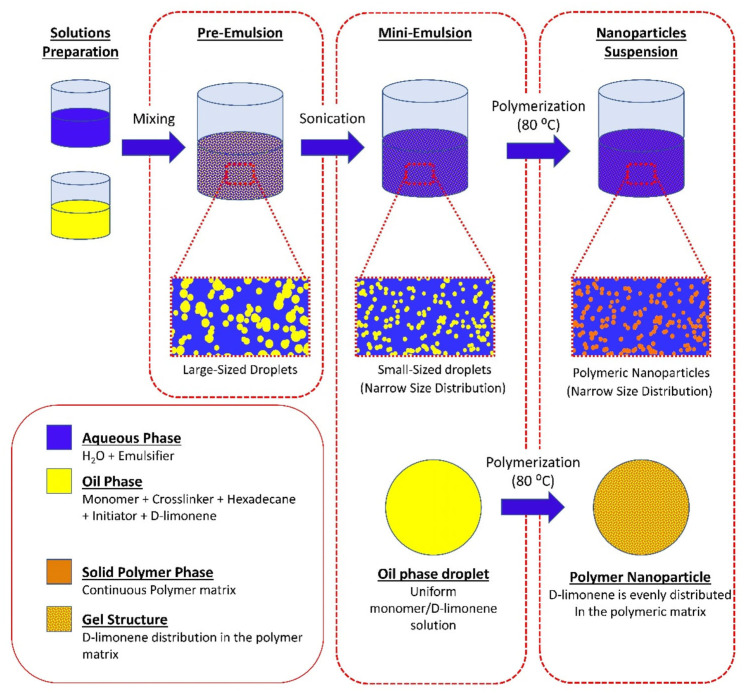
Schematic representation of the mini-emulsion polymerization process and the possible structure of the synthesized nanoparticles [54].

Citrus peels possess availability and nutrient-rich composition, including essential micro and macronutrients such as water, proteins, sugars, minerals, and EOs, leading to the consideration of being valuable matrices [50]. For example, the adsorption capacity of pomelo peel, a natural and versatile bio-absorbent, is able to adsorb epigallocatechin-3-gallate. This offers pomelo peel as a cost-effective and efficient option as a carrier for delivering natural products in functional food and dietary supplement contexts [51]. Eugenol and its derivatives (isoeugenol, methyleugenol, acetyleugenol, and eugenol oxide) from the *Cinnamomum tamala* leaf interact with lipid bilayers composed of palmitoyl-oleoyl-sn-glycero-3-phosphatidylcholine and palmitoyl-linoleoyl-sn-glycero-3-phosphatidylcholine, showing their antioxidant activity [52]. The antibacterial activity of EOs is often related to their hydrophobic nature, which can disrupt membrane integrity and function. Studies suggest that EOs affect bacterial cells’ external envelopes and cytoplasm through a series of events. While both Gram-positive and Gram-negative bacteria possess peptidoglycans in their cell walls, EOs have demonstrated potent effects against both types of bacteria, offering a promising alternative to traditional antibiotics [53]. Mini-emulsion polymerization offers advantages for producing nano-encapsulated systems, allowing for the development of stable, uniformly-sized droplets that retain their shape throughout the polymerization process [54]. Andriotis et al. used D-limonene in their research, a key component of citrus EOs with enhanced antimicrobial activity, as a model volatile compound to synthesize terpene-loaded NPs via mini-emulsion polymerization to optimize NP properties for potential applications in elevated temperature processes [54] (Figure 3).

EOs possess the synergistic effects of their compounds, leading to greater activity when applied as natural EOs compared to the sum of the effects of individual substances, while their qualitative and quantitative composition plays a crucial role in determining their antimicrobial potential [55]. The hydrophobic characteristics of EOs affect their direct integration into aqueous-based matrices and environments, potentially compromising their antimicrobial efficacy and applications. Higher concentrations may be necessary to attain desired functionalities in water-based matrices, potentially leading to changes in organoleptic properties and acceptability [26]. For example, the ideal carrier for oral DSs must be non-toxic and offer both chemical and physical stability for therapeutic proteins. Nano-sized colloidal systems are attractive candidates because they can encapsulate biomolecules, protect them from gastrointestinal degradation, and efficiently navigate through membranes related to hydrophobic/hydrophilic properties [56]. An oral matrix carrier is a self-ordered structure made of hydrophobically modified silica NPs, polysaccharides, biopolymers, and natural oils. Silica NPs attract amphiphilic protein molecules, which bind to them, while polysaccharides in the surrounding oil environment attach to the proteins. Natural oils (olive oil, sea buckthorn oil, or coconut oil) then encapsulate the active compound component, forming a protective complex that shields the protein from digestion and hydrolysis in the gastrointestinal tract [56].

Natural terpenes can be used as penetration enhancers for transdermal compound delivery. Terpenes, abundant in nature, offer promising advantages over synthetic enhancers, demonstrating higher enhancement activity and lower toxicity, making them preferable candidates for enhancing percutaneous compound absorption [57]. In subsequent sections, the most common terpenes and their delivery strategies will be discussed in detail.

### 4.1. Limonene Usage as a Penetration Enhancer

Orange, grapefruit, and lemon oils contain the main volatile component of citrus peel oil, commonly known as D-limonene [9]. Limonene oil belongs to the terpene family and has various biological properties, including anti-inflammatory, anti-cancer, antioxidant, and gastroprotective effects. It has potential as an adjuvant to modern therapeutic drugs in mitigating the progression of neurological diseases, i.e., limitation of nerve injury spread and reduction of hyperalgesia [58]. Limonene is widely used in DSs, providing drug release into the skin or transdermal release [11].

D-limonene nanoemulsion has been used as a carrier for curcumin insertion and transdermal absorption. Results indicate that the droplet size of the curcumin nanoemulsion influenced the cumulative concentration of its transdermal absorption, with larger droplet sizes showing higher steady-state flux. Additionally, this system showed significant potential as a carrier for transdermal DSs in food and cosmetic products [59]. The synthesis of polymeric NPs loaded with D-limonene demonstrated enhanced antimicrobial potency, suggesting their potential as promising materials for sterilization [54]. A self-microemulsifying DS was used to enhance the oral bioavailability of limonene, which was optimized for stability and release profile. Results demonstrated a significant improvement in oral bioavailability and the tissue distribution of limonene in the system compared to free limonene [60].

Transfersomes containing raloxifene hydrochloride have been optimized by limonene for transdermal delivery, varying phospholipid, sodium cholate, and limonene concentrations. An in vivo study showed that the optimized formulation improved the permeation of raloxifene into deeper layers of Wistar rat skin and significantly enhanced bioavailability compared to an oral raloxifene suspension [61]. A thermo-responsive intranasal limonene-based microemulsion mucoadhesive nano gel aimed to improve the efficacy of propranolol for managing migraine attacks. The nano gel exhibited good mucoadhesive properties, controlled release, and enhanced nasal permeability, leading to significantly improved propranolol brain availability compared to the control group, indicating its potential as a promising strategy for migraine management [58].

### 4.2. Eugenol Usage as a Penetration Enhancer

Eugenol, a phenol, is a key constituent of clove oil with anti-inflammatory effects, particularly in topical formulations [62]. However, the skin irritation associated with these formulations limits eugenol’s clinical use. To address this challenge, eugenol-loaded calcium citrate NPs (Eu-CaCit NPs) demonstrated biocompatibility and the ability to penetrate the dermis layer of intact human skin without cytotoxic effects. These findings suggest that Eu-CaCit NPs could be an effective carrier for the topical delivery of eugenol, offering a potential alternative for natural pain relief in topical applications [63]. Another example is that an O/W nanoemulsion formulation of eugenol significantly enhanced anti-inflammatory activity compared to a marketed piroxicam gel in a carrageenan-induced paw edema rat model. A higher concentration of eugenol did not correspond to higher anti-inflammatory effects. Additionally, a nanoemulsion containing piroxicam had diminished anti-inflammatory properties compared to formulations without piroxicam [62].

Eugenol microemulsions have been optimized for transdermal delivery of indomethacin, a model drug, by constructing pseudo-ternary phase diagrams using Tween 40 as a surfactant with or without ethanol or propylene glycol as co-surfactants. Results indicated that the presence of ethanol or propylene glycol increased the microemulsion zone. Higher eugenol concentrations and the co-surfactant presence enhanced indomethacin loading, release rate, and transdermal flux, with propylene glycol-containing systems showing superior performance [64]. Another DS was developed for resistant metastatic melanoma. Hyaluronic acid-coated liposomes loaded with a combination of anti-melanoma agents, including dacarbazine and eugenol, were developed using a solvent injection method guided by quality-by-design principles. This system showed higher cytotoxicity, increased late apoptotic cell numbers, and exhibited greater cell migration and proliferation inhibition compared to a dacarbazine solution. It is a promising strategy against aggressive melanoma that has reduced cytotoxicity and lower doses of chemotherapeutic agents [65].

### 4.3. Borneol Usage as a Penetration Enhancer

Borneol, a natural lipophilic monoterpenoid, has been extensively researched for its potential as a permeation enhancer across the blood–brain barrier, where it alters membrane lipid structures and modulates transport proteins. Additionally, borneol can improve drug delivery across various physiological barriers, including nasal and gastrointestinal layers, transdermal pathways, transcorneal routes, and the blood–optic nerve barrier [66]. Borneol penetrates the blood–brain barrier and modulates signaling pathways within the neurovascular unit. This component is a potential drug delivery agent for neurodegenerative disease therapies by regulating inflammatory and oxidative stress proteins [67].

Borneol has been evaluated as a matrix-forming component for in situ forming matrices (ISMs) targeting crevicular pockets. Drug-free and vancomycin hydrochloride-loaded borneol ISMs were compared. The borneol-based ISM demonstrated promising characteristics, such as low viscosity for improved injectability, sustained drug release over 14 days via a diffusion-controlled mechanism, and potent antimicrobial activity against various pathogens, suggesting its potential as an effective local treatment for periodontitis via crevicular pocket injection [68].

### 4.4. Menthol Usage as a Penetration Enhancer

Menthol, a cyclic monoterpene alcohol, contains cooling properties and has a lingering minty fragrance reminiscent of its source oil residues. This terpene is effective in enhancing the dermal penetration of pharmaceuticals [69]. At concentrations of 3.5% or lower, menthol altered the SCs’ original structure to varying extents, enhancing their fluidity and facilitating the permeation and retention of menthol. Two mechanisms were found: (1) menthol’s robust hydrogen-bonding capability allows it to compete for lipid-lipid hydrogen bonding sites, thereby weakening the stability of the skin lipid’s hydrogen-bonding network; and (2) menthol’s affinity for cholesterol, likely due to their similar molecular structures, suggests that its incorporation could enhance lipid membrane fluidity akin to cholesterol [70]. Xu et al. aimed to identify the impact of menthol on the permeability of dexamethasone disodium phosphate across the cornea and sclera in vitro. Rabbit eyes were subjected to topical drops and subconjunctival injections of dexamethasone disodium phosphate, both with and without the addition of 0.1% menthol. Results showed that concentrations of 0.05% and 0.1% menthol notably augmented the penetration of dexamethasone into the cornea. However, there was no observed alteration in dexamethasone penetration through the sclera and in the retina–choroid tissue under these conditions. Upon administration of topical drops containing dexamethasone with 0.1% menthol, a notable increase in dexamethasone concentration was observed in the cornea and aqueous humor tissues [71]. Another study by Krishnaiah et al. analyzed the impact of different solvent systems and concentrations of menthol on the permeation of ondansetron hydrochloride across rat epidermis. Solubility tests revealed the highest permeation rate with a 60% *v*/*v* ethanol–water system, prompting the preparation of hydroxypropyl cellulose gel formulations containing varying concentrations of menthol. Incorporating 8% *w*/*w* menthol into 2% *w*/*w* HPC gel resulted in optimal transdermal permeation of ondansetron hydrochloride, with a significant enhancement in drug flux observed [72].

Additionally, supramolecular eutectogels exhibit dynamic properties due to noncovalent interactions, making them responsive to stimuli, thus finding applications across various domains. The supramolecular interactions between the eutectic solvent components thymol–menthol and ethosuximide drug molecules are responsible for the gel structure and dynamics of the small molecules. In vitro release profiles, explored in different pH and temperature conditions, demonstrated the capability of eutectogel formulations to deliver ethosuximide over extended periods [73]. Encapsulating menthol within lipid-core nanocapsules using a nanoprecipitation method assesses its suitability for skin application. Results from the hen’s egg chorioallantoic membrane and cytotoxicity assays indicated the safety of topically applied nanocapsules, suggesting their potential as an aqueous solubility enhancer for menthol with improved thermal stability and as an alternative option for cosmeto-textiles with rapid dermal absorption properties [74].

### 4.5. Essential Oil Mixtures Usage as Penetration Enhancers

Terpenes, including carvone, 1,8-cineole, menthol, and thymol, combined with ethanol, enhance the percutaneous absorption of tamoxifen through the porcine epidermis in vitro. This mixture improved the partitioning of tamoxifen into the SC, suggesting potential applications for terpenes as enhancers for lipophilic drugs like tamoxifen [75].

In vitro and in vivo studies have identified the transdermal delivery of zidovudine using novel chemical enhancers, including t-anethole, carvacrol, thymol, and linalool, with L-menthol as a reference enhancer. An isopropyl alcohol/water solvent yielded better absorption than propylene glycol/water with most enhancers, indicating the potential of these enhancers for effective transdermal delivery of the drug [76].

The percutaneous absorption-enhancing effects of 11 monoterpenes ((+)-limonene; (-)-menthone; (+)-terpinen-4-ol; alpha-terpineol; 1,8-cineole; (+)-carvone; (-)-verbenone; (-)-fenchone; 9, p-cymene; (+)-neomenthol; and geraniol) were evaluated on the skin of hairless mice using three model drugs with varying lipophilicities: caffeine, hydrocortisone, and triamcinolone acetonide (TA). Terpenes applied at a concentration of 0.4 M in propylene glycol enhanced the permeation of caffeine, menthol, and geraniol, showing the most substantial increase. Additionally, terpenes improved the delivery of hydrocortisone, with both terpineols demonstrating the highest enhancement. However, the examined compounds did not impact the delivery of TA, suggesting their potential selectivity in the transdermal penetration of specific drugs [77]. In vitro and in vivo studies evaluated the permeation-enhancing effects of EOs extracted from *Alpinia oxyphylla* on indomethacin permeation through Wistar rat skin. Both AO-1 and AO-2 fractions significantly induced indomethacin permeation, with AO-2 exhibiting greater efficacy, which can likely be attributed to increased skin/vehicle partitioning. It also demonstrated limited skin irritation and differing effects on prostaglandin E(2) release from skin fibroblasts and lung epithelial cells [78].

Williams et al. demonstrated that α-pinene, 1,8-cineole, ascaridole, α-terpineol, and carvone have the ability to improve the skin penetration of the polar penetrant 5-fluorouracil (5-FU). Different terpenes showed varying levels of effectiveness; i.e., α-pinene, only modestly increased permeability, 1,8-cineole demonstrated a significant enhancement, suggesting a diverse range of activities among the tested compounds. The terpenes primarily increased drug diffusivity through the membranes via interactions with intercellular SC lipids rather than affecting drug partitioning or keratin interaction [79]. In addition, an in vitro study of poly(acrylic acid) gels incorporating 5-FU and tetrahydrogeraniol (THG) on rat skin showed that THG significantly induced the permeability of 5-FU. Higher THG concentrations increased permeability, indicating a potential application for THG in developing transdermal therapeutic systems for poorly absorbable drugs [80].

Tulsi and turpentine oil have been investigated as potential penetration enhancers for transdermal delivery of flurbiprofen. An optimized binary solvent mixture containing propylene glycol and isopropyl alcohol showed significantly higher permeation rates of flurbiprofen across rat abdominal skin compared to other solvent mixtures, and the addition of tulsi and turpentine oil further enhanced the flux of flurbiprofen [81]. Fang et al. identified the efficacy of sweet basil (*Ocimum basilicum*, OB) EOs as skin permeation enhancers and their potential for irritancy. Sweet basil EOs, containing various terpenes, notably improved the in vitro (keratinocytes and skin fibroblasts) skin permeation and deposition of indomethacin, with OB-1 exhibiting superior enhancement compared to OB-2, while showing minimal irritation in both in vitro and in vivo analyses [82].

Another study has demonstrated the potential of transferring some formulations’ development using eucalyptus oil to deliver ketoconazole through the skin. Results showed that EO improved the release and permeation of ketoconazole, demonstrating their ability to modify the skin barrier without undergoing any alteration themselves [83].

## 5. Release Mechanisms of Active Substances from Compound Delivery Systems

Delivery systems are designed to release encapsulated bioactive compounds in response to specific triggers, such as changes in pH, relative humidity, temperature, particle size, carrier system form, and properties of carrier materials and cross-linking agents. Effective encapsulation ensures the protection of bioactive compounds from external conditions until release. The release of encapsulated EOs from carrier systems is regulated by physicochemical mechanisms such as diffusion, swelling, melting, or degradation when exposed to environmental trigger conditions [26]. The topical application of EOs primarily involves skin penetration by interacting with the lipid constituents of the skin’s cell membrane. The depth of penetration depends on the chemical composition of the EO; e.g., oils like jojoba, avocado, soybean, and almond typically penetrate only the upper epidermis, while oxygenated terpenes can reach deeper layers of the skin. Certain oils (basil, tulsi, and tea tree) also can be penetration enhancers, both internally and topically; i.e., various mechanisms involve enhancing compound partitioning, disrupting the highly ordered intercellular lipid structure between corneocytes in the SC, and inducing conformational changes by interacting with intercellular protein domains [84]. Although progress has been made in designing degradable polymers for DSs, the successful translation of such materials is limited due to a lack of knowledge regarding the optimal combination of matrix properties for achieving controlled and sustained release. Poly α-esters are used for fabricating compound carrier matrices because they can degrade into harmless end products, and their degradation rates can be adjusted by modifying parameters like molecular weight, crystallinity, and backbone chemistry, making them versatile for various sustained release applications [85].

Despite significant advancements in diagnostic and therapeutic approaches for pulmonary disorders, effective treatment remains elusive. One of the primary challenges contributing to inadequate therapy is multicompound resistance, coupled with the difficulty of ensuring that active compound molecules reach the lower respiratory tract with minimal side effects. Research suggests that administering compounds directly to the lungs via inhalers may mitigate many side effects of oral or intravenous delivery [86,87]. When inhaled, EOs primarily act through two pathways: olfactory stimulation and respiratory stimulation. Through olfactory stimulation, they activate the olfactory nerve, transmitting signals to the brain. Their structural similarity to neurotransmitters and hormones allows them to stimulate olfactory chemoreceptors, triggering a cascade of physiological responses in the limbic and hypothalamic regions of the brain, ultimately inducing a sense of calmness and alleviating symptoms of anxiety and depression [84]. Due to volatility, EOs can access both the upper and lower regions of the respiratory tract through inhalation. Additionally, their antimicrobial and anti-inflammatory properties make them a potential option for effectively treating respiratory tract infections [88]. In addition to olfactory stimulation, EOs can modulate brain function by being absorbed through the alveoli. This absorption allows EO molecules to enter the bloodstream, traverse the blood–brain barrier, and potentially interact with specific brain areas. Through alveolar diffusion, volatile EO molecules may reach the systemic circulation and subsequently access the brain, particularly if they possess lipophilic properties, thereby eliciting positive psychological and physiological effects that alleviate mood disorders [84]. Over the past decade, inhalation aromatherapy has demonstrated benefits in addressing mood disorders like depression and anxiety. It has been observed that EOs can access brain tissue via the nasal–brain pathway, circumventing the blood–brain barrier. Once within the brain, they affect areas such as the cerebral cortex, thalamus, and limbic system, leading to the amelioration of anxiety and depression and enhancement of sleep quality [89].

Controlled release systems are developed for the specific active agent and designed to regulate compound exposure, facilitate passage through physiological barriers, protect against premature elimination, and direct the compound to its intended site of action, thereby reducing exposure elsewhere in the body and enhancing patient compliance. These systems can enhance the commercial value of existing compounds by prolonging patent protection and minimizing variability in compound product performance [90]. The main processes used for controlled compound release are dissolution, partitioning, diffusion, osmosis, swelling, erosion, and targeting (Table 2) [90].

### Compound Delivery Routes

The limitations of chemical enhancers due to their efficiency and safety, i.e., exhibiting poor permeation across the SC, cause compound release to the top layers, increasing the risk of skin irritation with higher concentrations [8]. EOs and their constituents are emerging as preferred alternatives due to their safety profile and ability to promote percutaneous absorption of compounds into deeper skin layers, facilitating the permeation of both hydrophilic and lipophilic compounds with low cytotoxicity. Documented by organizations like the Research Institute for Fragrance Materials and the National Toxicology Program, EOs demonstrate relatively low toxicity compared to synthetic penetration enhancers, suggesting their potential as safer alternatives for enhancing compound permeation through the skin [8].

Compound delivery through inhalation offers targeted delivery to the lungs, depending on pulmonary compound concentration, which reduces systemic active compound levels and results in higher pulmonary efficacy and fewer systemic side effects. However, the complex anatomy of the lungs and various pulmonary pharmacokinetic processes (deposition, dissolution, mucociliary clearance, adsorption to lung tissue, pulmonary metabolism, and systemic compound clearance) create challenges for effective compound delivery. The structural characteristics of the lungs, such as airway mucus acting as a barrier, can further impede compound retention and clinical effectiveness by trapping conventional compound delivery particles, necessitating the careful design of newer carriers with specific size and surface properties [86].

**Figure 4:**
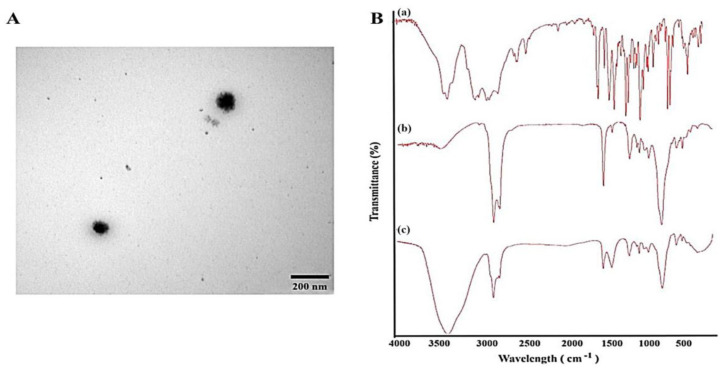
Example of EO-based microemulsion: propranolol-loaded limonene-based microemulsion system. (**A**) Transmission electron microscopical image of the optimized propranolol-loaded limonene-based microemulsion system, and (**B**) Fourier transform infrared spectra of (a) propranolol, (b) physical mixture of ME components, and (c) propranolol-loaded limonene-based ME [58].

Despite the advantages of nose-to-brain compound delivery, the smaller surface area of the nasal cavity limits molecule absorption, compounded by the inherent lipophilicity of the thin mucous lining, which favors only small, lipophilic species transport [58]. Recently, nanotechnology-based carriers have emerged as highly effective DSs capable of targeting multiple organs, shielding compounds from degradation, mitigating rapid clearance, and minimizing side effects. These carriers can be modified or surface-functionalized with various molecules to enhance targeting outcomes [86]. The design of compound nanocarriers loaded within thermo-responsive nanogels has become crucial for achieving the desired therapeutic effects in the brain. For example, microemulsions are promising due to their nanometric globule size and solubilizing properties, as well as their abilities to incorporate both lipophilic and hydrophilic compounds, enhance compound permeation through biological membranes, and prevent enzyme degradation. Additionally, the emerging concept of EO-based microemulsions presents a novel approach, leveraging the intrinsic properties of these oils to enrich the DS [58] (Figure 4).

Innovation in DSs aims to improve the bioavailability of active pharmaceutical ingredients. Oral delivery systems are preferred due to their diverse dosage forms, ease of administration, safety, and patient compliance. However, oral delivery systems face limitations such as poor compound stability in the gastrointestinal tract, first-pass metabolism, and solubility issues, particularly for peptide or protein-based compounds, leading to low bioavailability. To address these challenges, the transdermal route has emerged as a promising alternative, offering potential advantages such as non-invasive administration, improved patient compliance, and reduced disposal costs [76]. Ingesting EOs is not recommended for absorbing their therapeutic benefits as it is the least efficient method, with the oil typically passing through the digestive tract, including the stomach and small intestine, before reaching the bloodstream [104].

**Figure 5:**
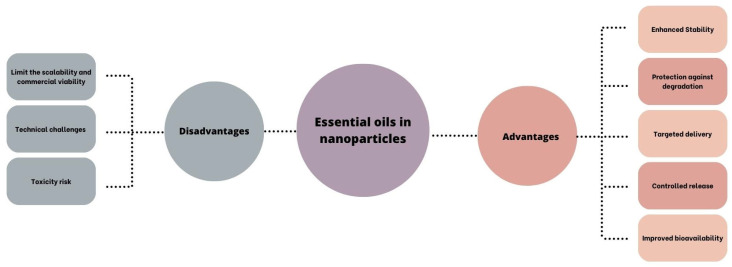
Advantages and disadvantages of the combination of EOs and nanoparticles [106].

To address such limitations of Eos as hydrophobicity, instability, volatility, and toxicity, encapsulation within delivery systems has emerged as a successful strategy. This approach enhances EO bioavailability, improves chemical stability, reduces volatility and toxicity, and enables targeted delivery to therapeutic sites, thereby enhancing patient compliance. Various strategies, including polymeric NPs and cyclodextrin inclusion complexes, have been explored for EO encapsulation, while lipid-based delivery formulations like micro- and nanoemulsions, liposomes, solid lipid NPs, and nanostructured lipid carriers have shown promise in this regard [5] (Figure 5). Although nanotechnology holds promise for therapeutic applications, it may cause risks to human health and the environment due to its physicochemical properties, dosage, route of administration, and interactions with cellular components. Factors such as particle shape, size, surface area, surface charge, interactions with proteins and lipids, and the ability to traverse biological barriers like the blood–brain barrier or placental barrier contribute to their toxicity. The pharmacokinetic characteristics of NPs play a crucial role in determining their pharmacological and toxicological behavior, with various mechanisms of toxicity including reactive oxygen species generation, lipid peroxidation, genotoxicity, membrane damage, protein denaturation, inflammation, granuloma formation, and disruptions in cellular functions such as lysosomal and mitochondrial dysfunction, apoptosis, and necrosis [105].

## 6. Discussion

Over the past five years, essential oils and their constituent terpenes have garnered increasing attention in the fields of medicine and pharmaceutical sciences due to their broad spectrum of biological activities, natural origin, and compatibility with human physiology. These volatile, aromatic compounds—primarily derived from plants—exhibit a wide array of therapeutic properties, including antimicrobial, anti-inflammatory, antioxidant, analgesic, anxiolytic, and anticancer effects. One of the key advantages of essential oils is their multifaceted mechanism of action. Such compounds as eugenol and menthol exert potent antimicrobial activity by disrupting microbial membranes, making them promising candidates for combating antibiotic-resistant strains. Linalool and limonene demonstrate notable anti-inflammatory effects through the modulation of pro-inflammatory cytokines and oxidative stress pathways, leading to their consideration in formulations targeting chronic inflammatory diseases. In oncology, in preclinical studies, monoterpenes such as d-limonene have shown the potential to induce apoptosis and inhibit tumor proliferation. In neuropharmacology, essential oil constituents like linalool, borneol, and α-pinene have been found to modulate the central nervous system, exhibiting sedative, anxiolytic, and neuroprotective effects, which have implications for treating disorders such as anxiety, depression, and neurodegeneration. To enhance the stability and bioavailability of EOs, various innovative delivery systems have been developed. Microencapsulation involves enclosing EOs within protective matrices, preserving their aromatic profiles and therapeutic properties while enabling controlled release. For example, liposome technology improves EO bioavailability by encapsulating both hydrophilic and hydrophobic compounds, ensuring targeted delivery and sustained release in cosmetic formulations. Self-emulsifying systems further enhance EO solubility and absorption, allowing their incorporation into diverse formulations. Overall, essential oils represent a promising, underexploited class of bioactive natural products with substantial potential in modern pharmacotherapy. Their versatility and biocompatibility make them attractive candidates for the development of novel therapeutic agents and delivery systems. As interest in natural and integrative medicine continues to grow, the role of essential oils and terpenes in pharmaceutical science is poised to expand significantly.

## 7. Conclusions

Since terpenes and essential oils (EOs) exhibit relatively low toxicity compared to synthetic penetration enhancers, they are potentially safer alternatives for improving compound permeation through the skin. This suggests their wider utilization in pharmaceutical formulations due to their safety profile and effectiveness. Integration into delivery systems may offer a promising approach to overcoming key limitations in drug delivery, thereby enhancing stability and bioavailability while facilitating improved penetration. As the pharmaceutical field continues to shift toward more natural, sustainable, and patient-friendly formulations, delivery systems for terpenes and EO are poised to play an increasingly important role. With continued innovation and rigorous scientific validation, terpenes and essential oils may become key components in the next generation of pharmaceutical therapies.

## Figures and Tables

**Table 2:** Processes of controlled compound release systems.

Process	Definition	Example
Dissolution	Process in which a substance (gas, liquid, or solid) becomes uniformly dispersed within a solvent to create a solution [91].	*Origanum syriacum* L. and *Lavandula hybrida* L. EOs have high dissolution rates, which correlate to their effectiveness in dissolving gutta-percha [92].
Partitioning	Drug (compound) solubilization within surfactants is driven by the partitioning of drugs into various regions of micelles, determined by the drug’s hydrophobicity, solubility, dissolution properties, partitioning area, and the ratio of sink condition [93].	Adding eucalyptus and mentha oils at a 15% concentration in DSs might retard the migration of trazodone hydrochloride (TZN) from the transdermal film into the SC, potentially elevating the drug’s saturation solubility within the polymer matrix and consequently reducing TZN flux. Adding EOs to the transdermal systems resulted in decreased flux values with nearly identical enhancement factors, while flux values following skin pretreatment showed a significant increase [94].
Diffusion	The movement of molecules from areas of higher concentration to lower concentration. For drug molecules (compounds) to diffuse through a polymeric medium, they must be in a dissolved, or mobile, state [95].	Franz diffusion cells experiment on mice with full-thickness skin revealed that Lippia origanoides, Turnera diffusa, eugenol, carvacrol, and limonene resulted in increased caffeine permeation, suggesting their potential efficacy as penetration enhancers in transdermal DSs [96].
Osmosis	Osmotic pumps offer advantages such as simplicity in operation without the need for electrical energy, leading to robust and easily miniaturized designs. Drugs within these pumps can be stored in either liquid or solid form, with the latter allowing efficient storage in a concentrated manner, occupying minimal space, and dissolution by water when needed for delivery as a liquid solution [97].	In vivo study on mice showed continuous administration of bergamot essential oil via an osmotic pump significantly mitigated neuropathic pain behaviors in mice, suggesting a sustained analgesic effect mediated by opioid receptors [98].
Swelling	Swelling occurs when the solvent penetrates the polymer [99].	Release of coriander EO containing microcapsules (chitosan, alginate, and inulin) followed a swelling-diffusion controlled process, depending on temperature and pH. Inulin reduces both encapsulation efficiency and the release ratio, while the release of coriander EO depends on a swelling–diffusion controlled process [100].
Erosion	Erosion can occur from the surface or the bulk of the polymer, or a combination of both, with control mechanisms including water and drug diffusion, swelling, chemical degradation, or dissolution of the polymer, potentially leading to zero-order release kinetics due to the interplay between these processes [95].	Menthol-based solid dispersion technique plays a critical role in controlling various physicomechanical properties for drug release, including swelling, erosion, and matrix stability. A study indicates that even though swelling decreased after 4 h, there was a corresponding increase in sulfamethoxazole release from the oral tablet matrix, suggesting that drug release in the later phase was controlled by erosion [101].
Targeting	The “targeting fraction” specifically binds with certain moieties or receptors at the target site, enabling personalized therapy with low drug dosage, high efficacy, and minimal side effects. Targeted nanocarriers are recognized for their ability to enhance drug bioavailability and efficacy through diverse targeting mechanisms, offering significant advantages in therapeutic outcomes [102].	The EOs exhibit cancer cell-targeting activity, enhancing the effectiveness of conventional chemotherapy drugs, and also demonstrate pro-immune functions when administered to cancer patients [103].

## Data Availability

Data sharing does not apply to this article as no datasets were generated.

## Abbreviations

5-FU	5-fluorouracil
CPE	chemical permeation enhancers
DS	delivery system
EOs	essential oils
Eu-CaCit NPs	eugenol-loaded calcium citrate nanoparticles
EβF	(E)-β-farnesene
ISMs	in situ forming matrixes
LPS	lipopolysaccharide
MeSA	methyl salicylate
NLC	nanostructured lipid carriers
NPs	nanoparticles
O/W	oil-in-water phase
OB	*Ocimum basilicum*
SC	stratum corneum
SNL	solid lipid nanoparticles
TA	triamcinolone acetonide
THG	tetrahydrogeraniol
TZN	trazodone hydrochloride
W/O	water-in-oil phase
